# The Modern Primitives: Applying New Technological Approaches to Explore the Biology of the Earliest Red Blood Cells

**DOI:** 10.1155/2013/568928

**Published:** 2013-10-03

**Authors:** Stuart T. Fraser

**Affiliations:** Disciplines of Physiology, Anatomy and Histology, Bosch Institute, School of Medical Sciences, University of Sydney, Medical Foundation Building K25, 92-94 Parramatta Road, Camperdown, NSW 2050, Australia

## Abstract

One of the most critical stages in mammalian embryogenesis is the independent production of the embryo's own circulating, functional red blood cells. Correspondingly, erythrocytes are the first cell type to become functionally mature during embryogenesis. Failure to achieve this invariably leads to *in utero* lethality. The recent application of technologies such as transcriptome analysis, flow cytometry, mutant embryo analysis, and transgenic fluorescent gene expression reporter systems has shed new light on the distinct erythroid lineages that arise early in development. Here, I will describe the similarities and differences between the distinct erythroid populations that must form for the embryo to survive. While much of the focus of this review will be the poorly understood primitive erythroid lineage, a discussion of other erythroid and hematopoietic lineages, as well as the cell types making up the different niches that give rise to these lineages, is essential for presenting an appropriate developmental context of these cells.

## 1. Historical Backdrop

Early in the 1900s, advances in microscopy and histology lead to a golden era of investigation into the processes regulating embryonic blood production. Pioneers such as Maximov, Sabin, and Jordan published a series of monographs describing blood cell production in the vertebrate embryo [1–3]. Much attention was focused on the unusually intimate relationship between developing endothelial cells and hematopoietic cells with the term hemogenic endothelium appearing at this time (reviewed in [4]). A distinct population of erythroid cells was identified and categorized as “megaloblasts”. These nucleated cells appeared to carry hemoglobin but were larger than the conventional anuclear red blood cells observed in the adult. These cells were first detected in the extra-embryonic yolk sac. Due to these characteristics, as well as their similarities to nonmammalian vertebrate erythrocytes, these cells were termed primitive erythroid cells [1] (which is often abbreviated to EryP). The distinctions between EryP and adult-type definitive erythroid cells (EryD) is the main focus of this review. The epithet “primitive” has proven to be somewhat distracting as hematopoietic stem cells with extensive self-renewing potential are also often referred to as being “primitive”. This review is focused on the primitive erythroid lineage originating in the yolk sac. This task however cannot be performed in isolation, and as such other blood cells and hematopoietic tissues will be discussed.

## 2. The Anatomy of Embryonic Blood Production

The hematopoietic system forms in several different anatomical locations including, within the embryo proper, the yolk sac, the placenta, as well as vitelline, umbilical and cranial blood vessels. The term conceptus is viewed by some as being old fashioned. However, considering the multitude of sites of hematopoiesis, the conceptus, which incorporates the extra-embryonic yolk sac, the allantois, chorion and placenta, and the embryo itself, is a useful descriptor for the collected structures in which blood cells are generated, expand in number, and then circulate. Here, I will very briefly outline the anatomical structures and regions critical to blood formation. This discussion primarily refers to the developing mouse embryo. Embryonic day of development (E) is used to identify developmental stages. For more complete descriptions of the regions of the conceptus which regulate blood cell production please refer to [5–9].

### 2.1. The Yolk Sac

The first site of hematopoietic development is the extra-embryonic yolk sac (YS) [6, 10]. This bilaminar membrane encapsulates the developing embryo proper and is composed of an outer layer of visceral endodermal cells forming an epithelial layer and an inner layer derived of extra-embryonic mesoderm. This mesenchymal layer will differentiate into blood vessels filled with hematopoietic cells [11, 12]. Hematopoietic cells first appear in a band towards the proximal end of the YS [13]. Histological analysis and assessment of marker protein expression of CD41 have reproducibly observed initiation of hematopoiesis in this “blood band” followed by expansion throughout the entire vasculature of the embryo [10, 13]. Similar observations were made using transgenic reporter mice [14, 15]. The YS histological structure changes rapidly in the few days following gastrulation. Clusters of mesoderm-derived vascular progenitors differentiate into the primary capillary plexus. This then expands throughout the YS. These vessels expand in number and complexity and differentiate into more mature vessels. Within the vessels, hematopoietic progenitors capable of giving rise to the myeloid and lymphoid lineages appear [16–18]. The first to differentiate are the primitive erythroid colony form cells (CFC) and the macrophage CFC [18]. Early megakaryocytes are also generated at this time [18]. Over the next few days of development, myeloid, lymphoid and eventually multilineage hematopoietic progenitors capable of repopulating myeloablated hosts arise in the YS [16, 17]. Hematopoietic activity in the YS ceases at approximately E12.5 though the mechanisms leading to this sudden loss of blood-producing activity are poorly understood. 

### 2.2. The Allantois, Chorion, and Placenta

The allantois, a sausage-shaped structure derived from the mesoderm, migrates through the exocoelomic cavity and fuses with the chorion to form the placenta [19]. The allantois and chorion, prior to fusion, are both sites of active hematopoiesis [20]. Isolated allantoic and chorionic tissues can generate erythroid, and myeloid cells *in vitro*. Notably, the erythroid colonies generated only expressed adult globin genes [20]. Following chorio-allantoic fusion, the placenta forms and invades the maternal tissue. Maternal trophoblasts form a complex vascular interface with rapidly developing vasculature originating from the conceptus. The placenta has recently become a site of hematopoietic development of considerable interest [21, 22]. Hematopoietic stem cells as well as erythroid, myeloid, and mixed colonies have been isolated from the mouse and human placentae. Mouse and human primitive erythroid cells are also thought to complete their maturation within the placenta [15, 23].

### 2.3. The Dorsal Aorta

The first site of intraembryonic hematopoietic cell development is thought to be the para-aortic splanchnopleura (Psp). This structure develops into the dorsal aorta (DA) at the level at which the gonads and mesonephros are forming (termed the AGM, aorta-gonad-mesonephros; reviewed in detail in [9]). The DA at E11.5–13.5 is a site where hemogenic endothelial cells give rise to hematopoietic stem cells [24, 25]. At the time of the HSC production in the AGM, the primitive erythroid cells are the dominant blood cell type filling the lumens of the rapidly expanding aorta. Over 10 million EryP are thought to be circulating at this stage of development [14, 26]. The HSC generated in the AGM migrate to the fetal liver where they give rise to the adult blood lineages. 

### 2.4. The Fetal Liver

The liver begins to develop as an outgrowth of the foregut at mid-gestation [27]. Almost as soon as it is histologically apparent, hematopoietic cells can be observed within this rapidly developing tissue [28]. By E12.5, the fetal liver is a conspicuously large, red tissue and is rapidly becoming the primary site of hematopoiesis. By E12.5, enucleated definitive erythroid cells are being released by the fetal liver and are replacing the EryP as the dominant red blood cell type in circulation. Macrophages are widespread throughout the parenchyma of the FL and interact with hematopoietic and erythroid progenitors to increase the output of mature, circulating blood cells. Failure to produce EryD due to genetic null mutation often leads to embryonic lethality at E13.5–14.5 indicating the developmental stage at which definitive erythropoiesis must take over from the EryP [29]. 

### 2.5. The Spleen and Bone Marrow

By E15.5, hematopoietic function of the FL decreases and the FL rapidly becomes a hepatic organ rather than a blood-forming tissue. Hematopoiesis then shifts to the developing spleen, and shortly before birth at day 20-21 hematopoietic cell production in the mouse migrates to its final location, the bone marrow. From there, hundreds of millions of mature, enucleated definitive red blood cells are produced daily. EryP are very rapidly diluted in the mass of EryD produced and are completely replaced by these adult-type red blood cells. 

## 3. Waves of Red Blood Cell Production

**Figure 1:**
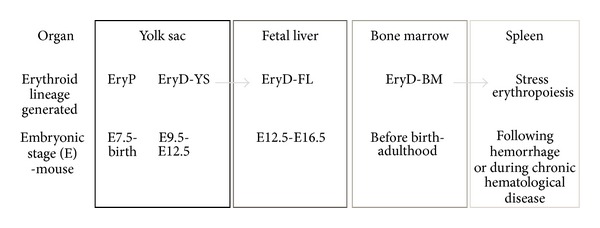
Distinct waves of red blood cell production (erythropoiesis) occur throughout ontogeny.

The independent production of the red cell component of the blood is a critical step in embryonic survival. Embryos will invariably die if they are incapable of forming their own circulatory system, including erythrocytes. The small, enucleated erythrocytes observed in healthy adult circulation are not the first blood cells to be generated. A number of different waves of erythroid cells are formed throughout mammalian embryogenesis (Figure 1). The first wave to appear is the primitive erythroid lineage, which is the major focus of this review. Cells forming this lineage appear shortly after gastrulation, are large, express embryonic globins, and circulate for much of their existence with a nucleus. Progenitors of these cells are found in the YS from E7.5 to E8.5, after which they migrate into the developing circulatory system and dominate the blood stream until the liver becomes a site of hematopoietic progenitor cell expansion and maturation (from E12.5 in the mouse fetus) [7, 8, 30]. 

From E8.5, a population of blast forming units-erythroid (BFU-e) cells can be isolated from the developing YS and shortly after in the bloodstream, embryo proper, and eventually the fetal liver [18]. A more mature population of colony forming units-erythroid (CFU-e) cells can be observed in the YS from E9.5, expanding in the bloodstream and expanding enormously in the liver from mid-gestation onwards [18]. These cells express adult globin genes and hence are termed “definitive”. A YS-derived definitive erythroid population has been identified with a remarkable capacity for expansion *ex vivo* in contrast to similar cells derived from the adult bone marrow [31]. These “extensively self-renewing erythroblasts (ESREs)” could be maintained as immature cells in culture for extended periods of time then induced to proceed through terminal maturation by the removal of dexamethasone or transferred to erythroid maturation medium [31]. Functional definitive erythroid cells can be found in the embryonic circulation from E11.5 [32]. Definitive erythroid progenitors cells enter the developing fetal liver by E11.5, prior to migration of multi-lineage hematopoietic stem cells, which are thought to be the origins of later-stage EryD. However, an unusual erythroid population was found to express one of the embryonic globins, *β*H1, but not the other embryonic *β*-globin gene, epsilon (*ε*). This YS-derived population could be observed in the fetal liver several days later. This population may be a “stop-gap” measure, supplying the required erythroid cell support before the mass production of definitive erythroid cells derived from hematopoietic stem cells in the fetal liver [31]. 

The fetal liver is a hematopoietic organ from the time it arises from the foregut at E10.5 until E16.5 when it reverts to being primarily hepatic. EryD derived from the fetal liver flood the circulation from E12.5. From this point onwards, the frequency of EryP rapidly declines as the primitive cells are no longer capable of dividing. EryD derived from the fetal liver (herein termed EryD-FL) are larger than their adult bone marrow-derived counterparts (which I will term EryD-BM). The BM is the primary site of blood production for the adult mammal. However, in the case of injury leading to hemorrhage, or in chronic erythroid diseases such as sickle cell anemia or *β*-thalassemia, the spleen can become a site of secondary erythroid cell production. This is referred to as stress erythropoiesis. BM-derived erythroblasts migrate to the spleen and give rise to another pool of erythrocytes to assist in the recovery from injury [33, 34]. 

## 4. Model Systems for Assessing the Processes That Regulate Blood Cell Formation in the Embryo

The primitive erythroid lineage is the first hematopoietic lineage appearing in mammalian development within initial commitment and development occurring at E7.5. At this stage of development, the mouse embryo is very small and only composed of several of thousands of cells, making dissection of primitive erythroid population very challenging technically. Whilst it is not impossible to isolate and purify the earliest committed EryP cells from the mouse embryo, studies of this nature require significant numbers of timed pregnant mice, large numbers of embryos, and well-trained researchers to perform the dissections [14]. Considering the challenges of cell number and the *in utero* development of eutherian mammalian embryogenesis, numerous alternative systems have been developed to study the first blood cells. 

### 4.1. Primitive Erythroid *In Vitro* Cell Culture

EryP colony forming activity was first observed by Wong and colleagues [35]. This group found that the early mouse YS contained two types of erythroid progenitor cell. One formed small colonies in methylcellulose in the presence of erythropoietin (Epo) alone and a second colony type required Epo and other cytokines to expand [35]. It was the former colony forming cell that gave rise to embryonic hemoglobin expressing cells [35]. This cell was termed the primitive erythroid colony forming cell or EryP-CFC, and can only be found in the YS from E7.5 to E8.5 [18]. Getting EryP, after the EryP-CFC, to mature *in vitro* has proven to be challenging. In the author's hands, EryP isolated from the E9.5 or E10.5 circulation divide several times but develop poor morphology and are often vacuolated and bi- or multinucleated. Enucleation is rarely observed. The conditions effectively modeling the microenvironment which EryP find themselves in during circulation require further investigation. Similar results were obtained from circulating EryP isolated from Syrian hamster embryos [36]. 

### 4.2. *In Vitro* Differentiation of Embryonic Stem Cells

**Figure 2:**
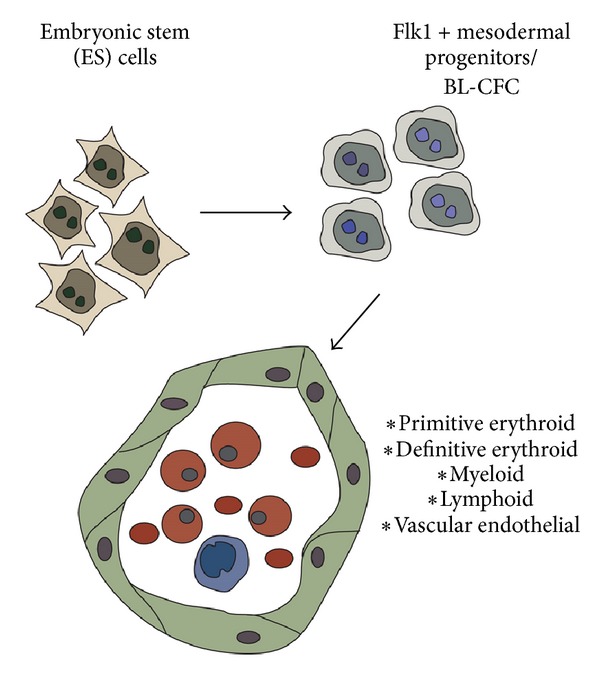
*In vitro* differentiation of embryonic stem (ES) cells into mesodermal progenitors of the hematovascular system.

Embryonic stem (ES) cells are immortal, undifferentiated cells derived from the inner cell mass of the developing blastocyst. ES cells have two remarkable properties that make them an excellent model for studying developmental processes. Firstly, they can be maintained in culture as undifferentiated, uncommitted stem cells as long as the soluble growth factor LIF (leukemia inhibitory factor) is present in the culture medium. Secondly, once LIF has been removed, ES cells will rapidly begin to differentiate and can give rise to all cells found throughout the body. If injected back into blastocysts, ES cells can contribute to all known tissues. *In vitro*, ES cells can be induced to differentiate into derivatives of all three germ layers (i.e., endoderm, ectoderm, and mesoderm) [37]. Depending on the culture conditions used, ES cells can be differentiated along the mesodermal lineage and have been recorded to give rise to the main cell types derived from the mesoderm, the blood, vascular and lymphatic endothelial cells, vascular smooth muscle cells, cardiomyocytes, and skeletal muscle [38–43]. The general differentiation pathway of ES cells into mesoderm derivatives is shown in Figure 2. There are numerous advantages of the ES cell differentiation system for modeling embryonic developmental processes. ES cell cultures can be scaled up to obtain larger numbers of rare intermediate and progenitor populations; the addition of soluble inhibitors or activators is straightforward, and the issue of embryonic lethality following null mutation of critical genes can be avoided. 

ES cells can be used to model the events leading to primitive erythroid cell appearance. EryP are derived from the mesoderm. Mesodermal-equivalent cells can be induced to differentiate from uncommitted ES cells by the removal of LIF and culture for 3 or 4 days in the presence of bone morphogenic protein or fetal bovine serum [38, 44, 45]. Flk1, the vascular endothelial growth factor-2, is a useful surface marker indicating mesodermal potential [46, 47]. Flk1-expressing cells purified from ES cultures at day 4 of differentiation can give rise to hematopoietic, endothelial, and smooth muscle cells [39]. Other groups have proposed that from day 3 of differentiation, a cell type termed the blast colony-forming cell (BL-CFC) is the origin of hematopoietic and endothelial cells [41, 48]. The BL-CFC is characterized by expression of the mesendodermal transcriptional regulator Brachyury, and BL-CFC can be purified according to expression of the Brachyury-promoter driven GFP construct [49]. Cells capable of generating EryP have been obtained from Brachyury-GFP-expressing cells from both the gastrulation stage embryo [50] and differentiating ES cell cultures [51]. BL-CFC; however, also express Flk1 suggesting that though differing terminology is used, these two systems are identifying overlapping, similar or possibly identical, progenitor populations. 

ES cells can differentiate into a range of hematopoietic cell *in vitro* if cultured under appropriate conditions [40]. Both BL-CFC and Flk1+ cells give rise to EryP-CFC [48, 52]. ES cells from the mouse [48], rhesus monkey [53, 54], and human [55, 56] have been used as a source of EryP-CFC and enucleated embryonic globin-expressing EryP. ES cells have also been used as a source of genetically altered EryP or progenitor cell lines. A random retroviral integration into ES cells lead to the generation of a cell line representing EryP-CFC termed EB-PE (embryoid body-derived primitive erythroid). EB-PE could give rise to EryP colonies *in vitro* and expressed embryonic globin genes [57]. This cell line required basic FGF and could be induced to differentiate in the presence of Epo [57]. HOX11 (now termed T-cell leukemia, homeobox 1, or Tlx1), is a homeobox transcription factor capable of transforming hematopoietic progenitors [58]. ES cells over-expressing HOX11 gave rise to a number of transformed cell lines with multi-lineage hematopoietic potential including EBHX11 which could give rise to both EryP and EryD *in vitro* [58]. 

### 4.3. Isolating EryP Progenitors and Monitoring Primitive Erythropoiesis

 EryP-CFC were initially obtained by preparing single cell preparations of the YS and culturing under appropriate conditions [35]. Maturing EryP, lacking progenitor activity, have been obtained from the embryonic circulation by dissecting mid-gestation or later stage embryos and exsanguinating the major vessels [59]. Another approach isolated RNA specifically from the developing EryP populations using laser capture microdissection (LCM). Plastic-embedded or frozen sections of the YS were stained with histological dye to allow ready identification of the different cell types in YS. By LCM, a small spot size laser pulses onto the section specifically removing a cell of interest, which is then collected individually, and RNA is extracted for gene expression analysis. Redmond and colleagues used this system to isolate mid-gestation EryP from the blood vessels of the YS and assess gene expression [60]. Comparative gene expression analyses of YS endodermal cells and EryP was performed by the same group [61]. 

Identification and purification of live EryP-CFC and EryP was a long-term goal of several research groups. A significant technical challenge was the lack of surface markers to distinguish them from other cells of the YS, or from the definitive erythroid population, that exist alongside the EryP. In the mouse, the paucity of monoclonal antibodies against erythroid-specific cell surface markers remains a significant technical impediment. The sole erythroid-restricted surface antigen in the mouse is recognized by the monoclonal antibody Ter-119 [62]. The antigen detected by Ter-119 is either Glycophorin A or an associated molecule [62, 63]. However, no specific mRNA has been isolated, and encoding Ter-119 and antibody binding appears to be dependent on glycosylation. Ter-119 is upregulated on the surface of EryP in the mouse once the cells have entered circulation [64]. Ter-119 expression therefore cannot be used to detect EryP earlier than E9.5. 

Another strategy to identify EryP in circulation was to capitalize on the presence of a nucleus as well as the differences in size and cellular granular contents between EryP and EryD [26, 65]. The use of the nucleic-acid binding dyes Hoechst or Vybrant Violet and the RNA-binding dye Thiazole Orange was used to isolate primitive and definitive reticulocytes from the E15.5 circulation [66]. These distinct types of immature enucleated cells expressed the characteristic globin genes confirming the origins from distinct arms of the erythroid system [65]. The same group used an antibody against embryonic *β*H1-globin to show that EryP do in fact enucleate. 

Flow cytometry using fluorescent proteins or antibodies is a very useful technology for monitoring EryP development and maturation. As mentioned, this was limited by a lack of specific cell surface antigens. To avoid this difficulty, transgenic mouse strains expressing enzymatic or fluorescent reporters were developed. The original transgenic line specifically labeling EryP was based on the lineage restricted expression of epsilon-globin (herein termed *ε*-globin) solely within the EryP [67]. Considerable effort had been put into resolving the regulator elements driving human embryonic *β*-like globin genes to determine lineage and stage-specific regulation [68–70]. A short human *ε*-globin gene promoter sequence and downstream micro locus control region (*μ*LCR) containing 4 DNAse hypersensitivity sites served as the template for the transgenes specifically labeling EryP [70]. The *ε*-globin::LacZ mouse was used to demonstrate the effect of adding Indian Hedgehog to explant cultures of pregastrulation embryos and to monitor the earliest appearance of committed EryP progenitors [71]. A transgenic line in which the *ε*-globin regulatory elements drove KGFP, a modified form of green fluorescent protein was used to monitor EryP during hematovascular development [71–73].

A second generation of transgenic mouse strains was generated in which a fusion protein composed of the histone H2B and GFP under the control of the *ε*-globin regulatory elements [28]. These lines generated a brighter fluorescent signal than the previous transgenic lines primarily due to the concentration of GFP in the nucleus by homing of the histone H2B portion of the fusion protein to the chromatin. This fusion proteins remain within the chromatin regardless of cell cycle stage and are useful in monitoring cell division, in contrast to earlier forms of GFP which were degraded during cell cycling [74, 75]. The *ε*-globin::H2B-GFP transgenic mouse lines were utilized to monitor enucleation of EryP as well as in embryo culture live confocal imaging systems [7, 14, 28] demonstrating the utility of this system. Highly purified EryP progenitors as well as maturing EryP were isolated using the *ε*-globin::H2B-GFP transgenic system offering precise biological replicates for transcriptome analysis, reducing the variability in the microarray data obtained [14]. A novel transgenic mouse line utilizing the same *ε*-globin regulatory elements driving cyan fluorescent protein (CFP) was very recently described [15]. The spectral separation between CFP and yellow fluorescent reporter (YFP) allowed the expression of both *ε*-globin::H2B-CFP and Flk1::H2B-YFP simultaneously with the same doubly transgenic embryo [15]. This allowed for the detection of both EryP and vascular endothelial cells in the same embryo. 

The technical advances described here have been instrumental in segrating EryP from surrounding cell types, allowing for the analysis of the developmental processes regulating the formation and maturation of this intriguing cell type. 

## 5. A Natural History of Primitive Erythroid Cells

Here, I will describe the critical stages of the life span of primitive erythroid cells from their appearance, expansion, migration, and through terminal maturation. Other vertebrates, including fish, amphibian and avian species are used to explore EryP biology; however, I will focus on knowledge gained from the mouse embryo. Loss of gene function has proven to be an invaluable tool for studying developmental biological processes. The development of the primitive erythroid lineage has also benefitted from this technology. Null mutant mice exhibiting EryP defects are described within the body of this review. The critical features of these mutants have also been summarized in Table 1. Likewise, the complex changes in surface marker expression throughout primitive erythropoiesis are summarized in Table 2. 

### 5.1. Commitment to the Primitive Erythroid Lineage

Identifying the cells that are to become EryP-CFC in the mouse embryo is extremely challenging due to the small number of cells, in a small tissue, which are capable of giving rise to this population. The posterior portion of the primitive streak, as identified by expression of the Brachyury-GFP transgene, contains a population that can give rise to embryonic globin-expressing cells upon culture [50]. This corresponds with the enrichment for cells which can give rise to EryP-CFC in the Flk1+ T-GFP+ population obtained from ES cell differentiation cultures at day 3.25 of differentiation [52]. In human ES cells, the promoter sequences of the mesendodermal marker *MIXL1* have been used to label primitive streak-like cells that can give rise to primitive erythroid cells with GFP [76]. Interestingly, over-expression of *MixL1* from the onset of ES cell differentiation induced an accelerated production of mesoderm and EryP-CFC confirming the role of this transcription factor in regulating the timing of hematopoietic induction including primitive erythropoiesis [77]. 

Gata1 is a transcription factor essential for hematopoietic development [78]. The regulatory elements from the *Gata1* gene have been used to drive GFP in the population committed to generate primitive erythroid cells. The Gata1-GFP transgene is expressed in the extra-embryonic mesoderm at E7.5 within the blood band. Isolated single Gata1-GFP+ cells can give rise to endothelial cells, definitive erythroid cells, and EryP [79]. The same transgenic reporter was used to identify mesodermal cells capable of giving rise to EryP and EryD in the *in vitro* differentiation of ES cell culture [80]. 

### 5.2. Appearance and Early Expansion of Primitive Erythroid Progenitor Cells

EryP are generated in the extra-embryonic yolk sac alone [18, 35]. Embryonic globin-expressing cells are first detected in the mouse YS shortly after gastrulation [10]. These cells are seen in close proximity to endothelial cells, suggesting a shared origin for these lineages [1]. The YS origin for EryP was elegantly demonstrated by the use of a null mutant mouse line in which Ncx1 sodium-calcium exchanger function was absent. Without Ncx1, the heartbeat could not be initiated, and migration of hematopoietic cells from the YS to the embryo proper could not occur [81]. EryP-CFC activity was not altered, though the presence of EryP was restricted to the YS in *Ncx1*-deficient embryos [81]. Concomitant with the appearance of EryP-CFC is the appearance of megakaryocyte and macrophage progenitors. A bipotent progenitor is present in the E7.25 YS and is capable of giving rise to both EryP-CFC and megakaryocyte colony forming cells (Meg-CFC) [82]. A similar cell type was identified during *in vitro* differentiation cultures of human embryonic stem cells [83]. 

As the vascular network expands throughout the YS, EryP proliferate within the newly formed vessels [14, 84]. The utility of the fluorescent transgenic reporter mouse strains came to the fore with live embryo time-lapse imaging of the migration of EryP throughout the developing YS vasculature [14]. EryP-CFC numbers increase from the appearance at E7.5 (late stage) and peak at 4–8 somite stage (approximately E8.25) [18]. The frequency of primitive erythroid cells early in embryogenesis has been estimated using the *ε*-globin::H2B-GFP transgene expression [14]. Shortly after gastrulation, at E7.5, the embryo contains between ~150–450 primitive erythroid cells. This number rapidly expands such that within 24 hours the frequency increases to 5,000, then nearly 9,000 and by the 6–10 somite stage approximately 11,000 GFP+ cells can be detected per embryo [14]. All EryP-CFC activity at E7.5 and E8.5 was restricted to the *ε*-globin::H2B-GFP+ population [14]. Globin gene expression is also increasing dramatically during these early stages of differentiation. *β*H1-globin is the predominant *β*-chain expressed at E7.5. *α*-globin is the predominant *α*-globin gene expressed although significant levels of theta globin transcript can also be detected [65]. Though expressed at considerably lower levels than *β*H1-globin, *ε*-globin is also being upregulated [14]. This is expression in turn is indicated by upregulation in the expression of the *ε*-globin::H2B-GFP transgene [14].

### 5.3. Surface Phenotype of EryP Progenitor Cells

Identification and purification of EryP-CFC at the single cell-level has been challenging due to a lack of specific surface markers. A number of surface antigens such as CD31, CD34, Flk1, the angiopoietin receptor Tie2, ESAM, and others are expressed by angioblasts and developing hematopoietic progenitors. EryP-CFC activity was also enriched in YS populations expressing the angiopoietin receptor Tie2 or PECAM-1/CD31 [85]. EryP-CFC activity was enriched by cell sorting of yolk sac cells expressing the integrin CD41 [13]. However, CD41 is also upregulated on definitive hematopoietic cells. Further enrichment of EryP-CFC activity was obtained when combining *ε*-globin::H2B-GFP expression with surface expression of either Tie2 or the stem cell factor receptor c-Kit [14]. Expressions of Flk1, VE-cadherin, CD41, Tie2, c-Kit, and CD31 on EryP-CFC were all confirmed using the *ε*-globin::H2B-GFP transgenic embryo. Flk1 and VE-cadherin were only detected on GFP+ cells at E7.5 and rapidly lost suggesting expression was persisting from the mesodermal precursor cells. EryP-CFC are obtained from the Flk1-negative population of combined YS/embryos at E8.5 [47]. Null mutation of Flk1 results in embryonic lethality with an absence of YS hematopoiesis [86]. Genetic tagging and tracing using doubly transgenic mice with a Flk1-Cre inducing a genetic label on all daughter cells via excision of a stop cassette in front of a LacZ reporter gene demonstrated that all hematopoietic cells, including EryP, are derived from Flk1-expressing progenitors [87]. 

### 5.4. Transcriptional Regulation of EryP Progenitor Activity

A number of transcription factors such as Lmo2, Scl/Tal1 and Gata2 are essential in initiating the hematopoietic programme including primitive and definitive hematopoiesis. The roles of transcription factors specifically within the EryP-CFC population are less clear. *Ldb1*-null mutant YS are devoid of erythropoiesis [88]. Similarly, *Ldb1*−/− ES cell cultures fail to form any erythroid cells. To avoid the mid-gestation embryonic lethality of *Ldb1*−/− embryos, *Ldb1* was conditionally deleted by generating *Ldb1*-floxed alleles that were excised in Tie2-Cre expressing cells. This resulted in embryos that generated EryP but failed to proceed through fetal liver erythropoiesis. However, primitive erythropoiesis in these mice developed abnormal morphology with enhanced mitosis and a higher frequency of immature basophils [88]. The transcriptional regulator c-Myc is also critical for EryP-CFC development. Mice lacking both copies of *Myc* die *in utero* and exhibited a profound paucity in EryP numbers [89]. The role of Class I Histone deacetylases in EryP-CFC activity was assessed by culturing YS explants with valproic acid (VPA) and determining EryP-CFC frequency. VPA treatment lead to a dose-dependent loss of EryP-CFC activity in YS explants suggesting that histone acetylase activity is required for primitive erythropoiesis [90]. 

### 5.5. Growth Factor Requirements of EryP Progenitors

The signaling molecules required for the expansion of EryP-CFC are gradually being identified. EryP-CFC require erythropoietin for growth *in vitro*; however, this hormone appears to regulate maturation more than progenitor activity [35]. Recent transcriptome analyses implicated transforming growth factor-beta (TGF-*β*) regulating in EryP-CFC activity [14]. The receptors for TGF-*β*, Acvr2b, Tgf*β*r1, and Tgf*β*r3 are expressed by EryP in the early YS. Growth of EryP-CFC in culture was inhibited by low doses (0.02 ng/mL) of TGF-*β*1. In contrast, higher doses (0.2 or 2 ng/mL) resulted in more than twice the number of colonies being generated. Neither TGF-*β*2 nor TGF-*β*3 had any effect on EryP-CFC activity [14]. Purified endothelial cells as well as the EryP themselves expressed the transcript for TGF-*β*1 suggesting both autocrine and paracrine signalling was taking place. Endoglin/CD105/Tgf*β*r3 is a coreceptor for TGF-*β* and is expressed by early EryP progenitors though lost rapidly during maturation [14, 85, 91]. Activin receptor IIB, which can also modulate TGF-*β* signaling, is also expressed by developing EryP [14]. Endoglin-deficient embryos and ES cell cultures show a profound reduction in EryP-CFC activity compared to wild-type counterparts [91, 92]. In contrast, over-expression of Endoglin on differentiating ES cell cultures leads to an enhanced production of EryP-CFC [92]. Gain-of-function of ALK1 results in an increase in EryP-CFC whereas over-expression of ALK5 leads to reduction in EryP-CFC numbers during ES *in vitro* differentiation cultures [93]. 

The Wnt signaling pathway is active in EryP-CFC [14]. This was observed in the genes upregulated in *ε*-globin::H2B-GFP+ cell transcriptomes and supported by positive immunoreactivity for EryP-CFC to antibody against activated *β*-catenin. A transgenic reporter of Wnt activity was also activated in hematopoietic cells within the blood band [14]. Wnt signalling in the mesodermal population that gives rise to EryP-CFC is also critical and is regulated through the intermediary protein Numb. Activation of the Notch signalling inhibits EryP-CFC frequency specifically without significantly impacting definitive hematopoiesis [94]. Furthermore, loss of a single allele of the Notch ligand delta-like 4 resulted in a significant reduction of EryP-CFC activity in heterozygous embryoid body *in vitro* differentiation and from heterozygous YS [95]. In contrast, definitive erythropoiesis was unaffected by delta-like 4 heterozygosity suggesting that this defect was more profoundly altering primitive erythropoiesis [95]. 

Vascular endothelial growth factor (VEGF) prevents primitive erythroid cell apoptosis [96]. VEGF-hypomorphic embryos lack morphologically normal EryP. Culture with VEGF leads to an increase in EryP cell size as well as a dose-dependent increase in EryP-CFC numbers generated from embryonic stem cells *in vitro* [96]. In a technical tour de force, Drogat and colleagues explored the effect of deleting or over-expressing VEGF specifically within the erythroid compartment. The deletion of VEGF164 isoform by the EpoR-Cre transgene resulted in enhanced and accelerated EryP differentiation [97]. Deletion of VEGF164 in EryP did not result in changes in EryP-CFC frequency. However, over-expression of VEGF164 resulted in an approximate 50% reduction in EryP-CFC frequency. This reduction in EryP-CFC could be rescued by over-expression of the erythroid transcription factor Gata2 specifically within the erythroid compartment [97]. The production of VEGF164 by EryP-CFC results in autocrine signaling affecting the EryP-CFC themselves. Transcripts for VEGFB were also detected in EryP in the early YS though rapidly lost upon initiation of circulation [14]. 

### 5.6. The Primitive Erythroid Progenitor Niche

The niche that EryP-CFC reside in supply other essential soluble factors required for the survival of EryP-CFC. c-Kit and Tie2, expressed by EryP-CFC are receptors for stem cell factor (SCF) and angiopoietin 1 (Ang1), respectively [14]. Transcripts encoding these soluble factors are present in purified populations of YS endothelial cells (for SCF) and endothelial cells and visceral endoderm cells (for Ang1) [14]. The EryP-CFC in turn produce the transcripts encoding a number of soluble factors including Insulin-like growth factor 2 (IGF2) and macrophage migration inhibitory factor (MIF). Intriguingly, MIF has been implicated in suppressing erythropoiesis [98]. However, our current state of knowledge is limited to RNA transcription detection of MIF in EryP, and no protein expression has been reported.

Oxygen partial pressure regulates EryP-CFC activity. Culturing purified *ε*-globin::H2B-GFP+ cells isolated from the early YS in hypoxic (2–5% O_2_) compared to atmospheric oxygen (21% O_2_) resulted in a 2.5-fold increase in EryP colony formation [14]. The size of the EryP colonies was also larger when cultured in hypoxic conditions. YS erythropoiesis at E8.5 is greatly reduced in the absence of the hypoxia mediator ARNT [99]. EryP-CFC also express an unusual range of hypoxia-related genes including metabolic genes associated with the Warburg effect. EryP-CFC express the transcripts for Glut1 and Glut3 glucose transporters. These are directly regulated by hypoxia. Similarly, expression of the Warburg effect regulators Pgk1 and pyruvate kinase M2 in EryP-CFC was elevated under low oxygen partial pressure [14]. 

EryP-CFC express *α*4*β*1 integrin which is a receptor for vascular cell adhesion molecule-1 (VCAM-1) [14]. Sturgeon and colleagues made the recent intriguing observation that VCAM1-expressing cells from *in vitro* differentiation cultures of ES cells induce maturation of EryP-CFC into a more mature EryP population. These findings came from a study of microRNAs expressed during ES cell differentiation to EryP-CFC. miRNA-126 was found to be upregulated during this process. Over-expression of miRNA-126 lead to more EryP-CFC in the differentiation cultures, whereas null mutation of miRNA-126 lead to loss of EryP-CFC and an increase in maturation surface markers on the EryP [100]. VCAM1 is expressed by a mesenchymal population, distinct from EryP and endothelial cells. Coculture of EryP-CFC with this mesenchymal cell type lead to accelerated maturation of EryP-CFC into EryP [100]. These findings would have been strengthened by assessing the location of VCAM1+ cells in the YS, however they offer intriguing insight into how the YS niche regulates the EryP-CFC activity.

### 5.7. EryP Transition from YS-Residence to Circulation

EryP-CFC in the early YS express a range of adhesion molecules including integrins *α*4, *α*5, *α*6, *β*1, and *β*3, as well as CD41 and CD44. Surface expression of these proteins decreased rapidly between E8.5 and E9.5 [14]. This window of differentiation corresponds to the sudden loss of EryP-CFC activity, the initiation of circulation and placentation. Transcripts of other molecules that are significantly downregulated during this transition from sedentary progenitor to circulating functional oxygen-transporting erythroid cell includes transcription factors such as Gata2 and Lmo2, Hmga1; signaling molecules related to Igf2 signaling; and glucose metabolism regulators Pkm2 and enolase-*α* [14]. Collectively, in the 24-hour window of development between E8.5 and E9.5, all EryP-CFC activity is lost, metabolism changes significantly; and a number of growth factor receptors and adhesion molecules are simultaneously lost from the cell surface [14]. This results in the release of dividing, functional primitive erythroid cells into the the embryonic, extraembryonic, and placental blood vessels, delivering O_2_ and removing CO_2_ from 10 developing embryonic tissues. 

## 6. Primitive Erythroid Cells in the Embryonic Circulation

By E9.0, the embryonic heart has begun beating, the blood vessels connecting the embryo, placenta and yolk sac are all in place, and circulation has commenced [8, 84, 101]. The presence of EryP in different locations within the conceptus has served as an indicator of the commencement of circulation [84, 102]. As the embryo matures, the circulation is dominated by EryP, from E10.5 until E13.5, when EryD originate from the fetal liver and become the dominant erythroid cell type in the bloodstream [26, 64]. During their period in the bloodstream, EryP divide up to 6 times. At E12.5, the circulation is still dominated by EryP that are estimated to the number of approximately 12 million cells; however, cell division has largely ceased as the nucleus has condensed [26]. Transcriptome analyses of EryP at each developmental day from E7.5–E12.5 reveal profound changes in the types of genes expressed as the EryP mature. However, the period during which the EryP are in circulation (i.e., E9.5–E12.5) is characterized by a drop in the number of different transcripts detected. In other words, the circulating EryP become relatively stable in the number of genes they are expressing in contrast to early stages and the final stages of terminal differentiation [14]. 

### 6.1. Cell Surface Phenotype

The initiation of circulation, as well as placentation, and the movement of EryP throughout the developing embryo coincide with the loss of EryP-CFC activity in the YS as well as the rapid loss of numerous adhesion molecules from the surface of EryP [14]. In contrast, once in circulation EryP upregulate the expression of Ter-119 [64]. The combination of CD71 and Ter-119 is a useful system to segregate the progressive stages of definitive erythroid development. CD71 is upregulated initially, followed by co-expression with Ter-119 at the erythroblastic stage and loss of CD71 during maturation from reticulocyte to definitive erythrocyte [103]. Using the *ε*-globin::KGFP mouse we were able to determine that circulating EryP progress through the same developmental pathway [64]. Circulating EryP at E9.5 express high levels of CD71, then by mid-gestation, Ter-119 was upregulated, and by E12.5, CD71 was downregulated [64]. We also found that a range of other surface markers is expressed by circulating EryP including the GPI-anchored molecules CD24 and CD55, as well as CD147, also known as basigin [64]. The tetraspanin CD9 is also expressed on the surface of circulating EryP and is a useful discriminator of EryP and EryD in the embryonic bloodstream [104]. The erythropoietin receptor (EpoR) is required for the maturation of EryP in circulation. EpoR-deficient embryos exhibit normal EryP differentiation and frequency at E9.5 however their number rapidly falls by mid-gestation with a proliferation defect [105]. 

### 6.2. Nuclear and Cytoplasmic Changes in Circulating EryP

Profound changes in nuclear structure and architecture take place in EryP as they mature semisynchronously in the embryonic bloodstream [64]. Between E9.5 and E12.5 the nuclear : cytoplasm volume ratio reduces to almost 1/5th of its initial volume [64]. Nucleoli are clearly abundant in E9.5 EryP. However, one day later, nucleoli are absent [64]. This is, at least in part, regulated by the transcriptional regulator c-Myc. Conditional deletion of *Myc* results in EryP that condense their nuclei several days earlier than wild-type EryP [106]. These mutant primitive erythroblasts also fail to proliferate in circulation [106]. 

The nucleus condenses as maturing EryP circulate. Proliferation rate and cell cycle simultaneously decrease. At day 10, most EryP are cycling, however this rapidly slows from E11.5 onwards. Mitotic cell number decreases rapidly [107]. RNA content, synthesis, and processing are all greatly reduced by E13.5 [108]. By day 13.5, EryP are no longer proceeding through the cell cycle [15, 109]. Chromatin remodeling is active in circulating EryP. Deletion of the chromatin-remodeling enzyme Brg1 in embryonic Tie2-expressing cells leads to embryonic lethality by mid-gestation [110]. Circulating EryP lacking Brg1 are highly fragmented with abnormal cell morphologies [110]. Likewise, null mutation of the SWR1-class ATPase mDomino (also known as p400) resulted in a severe defect in EryP maturation with reduced erythroid gene expression [111]. Embryos lacking the enzyme linked to ubiquitination and posttranslational modification of proteins, Ufm1, exhibit abnormal EryP maturation with unusual *ε*-globin-expressing multi-nucleated cells in circulation [112]. 

Large-scale changes in the cytoskeleton during EryP maturation occur at the same time as pronounced changes in nuclear structure and gene expression. The loss of intermediate filaments, along with changes in the actin cytoskeleton, lead to the nucleus becoming “free-floating” and eccentric within the EryP. Early EryP possess intermediate filaments. The major protein forming the erythroid IF network is vimentin. Early circulating EryP express vimentin and possess clearly identifiable IF [109]. However, as EryP mature in circulating, vimentin expression is lost [109]. The actin-binding protein gelsolin is expressed by both EryP and EryD. However, gelsolin-deficient EryP remain nucleated throughout development and circulate as late as E17.5 as nucleated cells [113]. Gelsolin is expressed in erythroid cells from many different species, is thought to cap actin filaments and assist in the formation of the actin-spectrin-adducin based cytoskeleton typical of erythroid cells [114]. The cytoskeleton of erythroid cells also requires erythroid tropomodulin expression. EryP express the cytoskeletal regulator erythroid tropomodulin (E-Tmod) [115]. The mechanical strength of *E-Tmod*-deficient EryP is reduced compared to wild-type EryP [115]. 

### 6.3. Transcriptional Regulation of EryP Maturation in Circulation

Null mutation of the transcription factors *Eklf* (also known as Klf1) and *Runx1* result in EryP with altered membrane stability and phenotype. Surface expression of CD71, CD9, CD55, and CD147 is dysregulated in embryos lacking both copies of the transcription factor *Eklf* [104] with slightly lower expression of CD9 and CD55 and slightly higher levels of CD147. Strikingly, Ter-119 is completely lost from the cell surface of *Eklf*−/− erythroid cells [116]. Interestingly, in spite of being critical in hemoglobin gene regulation, *Eklf*-deficiency did not significantly alter *ε*-globin::H2B-GFP transgene expression [104]. This allowed us to observe that loss of a single allele of *Eklf* results in loss of Ter-119 expression specifically on EryP [104]. *Eklf*−/− EryP appear to have significant defects in membrane stability as well as reduced expression of genes critical to erythroid maturation [116, 117]. Bizarrely, circulating *Eklf*-deficient EryP carry CD31 and CD41 on their surfaces, similar to earlier staged EryP. This occurs even though erythroid maturation markers are upregulated normally resulting in maturing EryP in circulation expressing progenitor-stage surface markers. CD24 is delayed in its expression in EryP lacking *Eklf * [104]. CD24 expression is also reduced on EryP lacking another KLF family, *Klf2* [118]. *Klf2*-deficient EryP show irregularly shaped cell membranes with unusual pseudopodia [119]. Compound null mutation of *Eklf* and *Klf2* resulted in even more severely altered EryP morphology with cytoplasmic blebbing, unusual nuclear morphology, deficiency in nuclear condensation, and frequent binucleation [120]. In contrast, *Runx1*-deficient EryP show a less severe alteration in EryP morphology. *Runx1*-null EryP show reduced levels of Ter-119 surface expression, abnormal “dimples” in their cell membranes, and slight membrane instability [121]. This less severe phenotype suggests that *Runx1* is a downstream target of *Eklf* in EryP. 

Several other transcriptional regulators are critical in this stage of primitive erythropoiesis. Gata1 is a hematopoietic-specific transcription factor that regulates EryP maturation. Null mutation of *Gata1* results in normal YS erythropoiesis but arrest of EryP maturation during circulation [122]. Mice lacking both *Gata1* and *Gata2* essentially lack all primitive erythropoiesis [123]. Complementation of the loss of *Gata1* with over-expression of *Gata4* does not completely ameliorate the *Gata1*-deficient phenotype. Terminal EryP maturation is impaired in *Gata4*-rescue and *Gata1*-deficient (G4R) mice with larger, less condensed nuclei at E12.5 compared to wild-type EryP [124]. At E14.5, G4R EryP are pyknotic [124]. The effects of the different domains of the Gata1 protein have been explored using transgenic complementation of *Gata1*-deficient mice. Primitive erythropoiesis requires the C-terminus zinc finger of Gata1. Interestingly, expression of the N-terminus zinc finger was enough to rescue primitive but not definitive erythropoiesis in *Gata1*-deficient mice [125]. *Gata2*-deficient EryP appear to be normal [126]. Null mutation of *Myb* results in defective definitive erythropoiesis but has no obvious impact on primitive red blood cell formation or maturation [82, 127]. Null mutation of *Erg* likewise had no impact on EryP [128]. Mice lacking both copies of the histone methyltransferase *DOTL1* show reduced EryP-CFC frequencies; however, from the relatively basic analysis performed, circulating EryP appeared normal [129]. Embryos lacking both copies of *RBPkj*, an adaptor protein transducing Notch signals, show an increase in Ter-119+ cells derived from the YS at E9.5 with decreased levels of apoptosis observed [130]. 

### 6.4. Hemoglobin Metabolism in Circulating EryP

Hemoglobin production escalates in circulating EryP [131, 132]. The recently reported null mutation of the *α*- and *β*-globin genes in developing mouse embryos, not surprisingly, results in embryonic lethality at E13.5 [133]. The targeted deletion of the embryonic globin genes has not been reported. However, ablation of the adult globin genes did result in changes to EryP maturation. This is presumably due to the small amount of adult globins which are expressed in EryP. Hb-deficient EryP were microcytic and immature in development compared to wild-type counterparts with the authors concluding that these Hb-deficient EryP are essentially thalassemic [133]. As EryP mature in circulation, their pattern of globin gene expression changes. In particular, epsilon globin is upregulated compared to the other critical embryonic *β*-globin, *β*H1. By E15.5, the predominant globins present in EryP are *α*-globin and *ε*-globin [65]. This requires extensive upregulation of the heme metabolism machinery [14]. One enzyme which is critical to the production of heme in erythroid cells is erythroid 5-aminolevulinate synthase (ALAS-E). This gene is regulated by both Gata1 and cellular iron availability. Null mutation of *ALAS-E* resulted in embryonic lethality at E11.5 and exhibit arrest of EryP at an immature stage [134]. Abnormal iron accumulation was observed in *ALAS-E*-null EryP at E9.5, and the transferrin receptor CD71 was significantly reduced in expression [134]. Expression of human ALAS-E in mice lacking endogenous ALAS-E resulted in primitive erythroid ring siderocytes formation [135]. CD71 expression on rescued EryP was improved compared to *ALAS-E* deficient EryP [135].

The orphan receptors TR2 and TR4 are thought to suppress the expression of embryonic globin genes. TR2/TR4 transgenic over-expression in erythroid cells leads to a depletion in the number of EryP-CFC and circulating EryP [136]. However, this is not an embryonically lethal defect as fetal liver definitive erythropoiesis is normal, and a Mendelian ratio of transgenic mice is achieved at birth [136]. This suggests that the transient anemia seen in these embryos is due to a paucity of EryP that can be rescued by definitive erythropoiesis. Sox6 is also a known repressor of embryonic globin gene expression and silences epsilon globin expression in the definitive erythroid lineage [137]. *Sox6*-deficient embryos maintain a population of nucleated, embryonic globin-expressing cells in their circulation until birth which, the authors claim, are unusual definitive erythroid cells [137]. 

Gata1 regulates the transcription of a series of genes critical to EryP survival [78]. The mitochondrial transporter ABC-me (encoded by *ABC10*) is directly regulated by GATA-1 and is thought to direct the transport of heme intermediates out of the mitochondria, thus preventing heme toxicity [138]. Embryos lacking *ABC10* die *in utero* shortly after gastrulation [138]. *ABC10*-deficient EryP at E10.5 are poorly hemoglobinised, fail to upregulate CD71 and Ter-119, are apoptotic, and show high levels of mitochondrial oxidative stress [138]. 

## 7. Terminal Maturation of Primitive Erythroid Cells

By the time they have reached the liver, the frequency of EryP will have expanded in number by 30,000-fold [14, 26]. Towards the end of their period in circulation, nucleated primitive erythroblasts upregulate a series of adhesion molecules including *α*4, *α*5 and *β*1 integrins, and CD44 [64]. 

### 7.1. EryP Enucleation

The most pronounced change in EryP morphology, as a part of terminal maturation, is the expulsion of the condensed nucleus to produce a circulating primitive reticulocyte. EryP from Tammar Wallaby (*Macropus eugenii*) and Gray Short-tailed Opossum (*Monodelphis domestica*) embryos showed hemoglobinized cells the size of marsupial EryP circulating without a nucleus [139]. Further evidence for enucleated EryP also came from studies of Syrian hamster embryogenesis [140]. Kingsley and colleagues showed the progressive reduction in size of maturing EryP alongside the appearance of enucleated cells larger than fetal liver-derived EryD that expressed embryonic globin [26]. These findings were supported using a combination of the *ε*-globin::KGFP transgenic embryos and the DNA-binding dye DRAQ5 [64]. DRAQ5 is a synthetic anthrocycline dye that rapidly crosses the cell membrane and is brightly fluorescent upon binding to nucleic acid. The *ε*-globin::KGFP transgene was used to identify EryP as EryD numbers increased rapidly, whereas DRAQ5 identified the presence or absence of the nucleus. Enucleation could be quantified using this combination by flow cytometry. The peak of enucleation was observed at E13.5–14.5 [26, 64]. 

Morphometric analysis of maturing Syrian hamster EryP revealed the presence not only of enucleated EryP but also of “naked nuclei” in the peripheral blood, presumably arising from EryP [140]. Enucleation in the mouse embryo was found not to require splenic pitting or karyolysis [141]. Few EryP were observed in the developing spleen whilst a large number could be detected in the vessels of the fetal liver [141]. Free nuclei could be observed in circulation. These consisted of the condensed nucleus surrounded by a thin rim of cytoplasm and were termed “pyrenocytes”. The thin rim of cytoplasm in the pyrenocytes contained embryonic globin implying an EryP origin. These findings were strengthened by observations made using the *ε*-globin::histone H2B-GFP transgenic mouse which specifically labels the chromatin with the green autofluorescent reporter [28]. The nucleus of EryP, regardless of being within or outside of the cell, could be monitored by flow cytometry, confocal, and live cell imaging. H2B-GFP+ cells were clearly observed within both vessels and parenchyma of the fetal liver with H2B-GFP+ cells sorted from the fetal liver showing typical EryP morphology and embryonic globin expression [28]. 

### 7.2. EryP Interactions with Fetal Liver and Placental Macrophages

Maturing EryP in the circulation and FL upregulate adhesion molecules and interact with FL macrophages [28, 141]. Blockade of erythroid *α*4*β*1 integrin interactions with VCAM-1 present on FL macrophages reduced EryP binding [28, 141]. EryP form multicellular clusters with fetal liver macrophages termed erythroblastic islands [142]. These clusters are thought to supply developing erythroblasts with iron and other nutrients as well as to engulf and destroy the expelled nuclei or “pyrenocytes” [142]. Single nuclei could be observed in fetal liver macrophages cultured together *ex vivo*. Degrading *ε*-globin::H2B-GFP+ nuclei could also be observed in fetal liver macrophages *in vivo* [7, 28]. With the fluorescent histone tags to monitor nucleus dynamics, we could assess presence of surface membrane proteins on the expelled nuclei. We found that adhesion molecules including CD44 and *α*4*β*1 integrin were preferentially partitioned onto the surface of the thin rim of cytoplasm surrounding the expelled nucleus. This presumably results in a more adherent pyrenocyte, more easily engulfed by the macrophage bearing receptors for these adhesion molecules, as well as generating a less adherent primitive reticulocyte free to reenter the embryonic circulation [28]. Correspondingly, enucleated EryP fail to form EBIs with macrophages in *ex vivo* EBI “reconstitution” experiments [28]. EryP in *Eklf*-null fetal livers is abnormal. All adhesion molecules examined were expressed at lower, less consistent levels compared to wild-type FL EryP, or were absent [104]. 

CD9, a tetraspanin expressed widely throughout the body, was observed to be restricted to the primitive erythroid lineage at a time when both red blood cell populations are circulating simultaneously. Indeed, CD9 expression in combination with the erythroid-specific antigen Ter-119 served as specific markers to separate EryP and EryD from the later stage circulating population [104, 143]. As soon as enucleated was achieved, all CD9 expression was lost, suggesting that CD9 was partitioned onto the membrane surrounding the enucleation EryP cell and disposed of from the cell surface [104]. Analysis of EryP nuclei labelled with the *ε*-globin::H2B-CFP transgene showed that placental macrophages also engulf and destroy primitive erythroid nuclei [15]. This process appears to be occurring more frequently in the fetal liver though it is now clear that several highly vascularized sites of the conceptus, bearing macrophages, are capable of engulfing and clearing primitive erythroid nuclei. 

### 7.3. Primitive Reticulocytes and Clearance

Enucleated EryP do not bind to FL macrophages and lack expression of the adhesion molecules seen on nucleated EryP in the FL [28, 64]. This suggests that following enucleation, EryP can be released into the circulation as reticulocytes. Indeed, large enucleated EryP can be detected in circulation [26, 64, 131, 140]. Between E14.5 and E17.5, EryP lose approximately 35% of their surface area and 50% of their total volume [143]. Circulating enucleated EryP express higher levels of Ter-119 and lower levels of CD71 whilst CD147 expression is unchanged [64]. Concomitantly, the stiffness of the EryP cell decreases as the cell matures, indicated by an increase in cellular deformability, possibility due to a loss of actin and tubulin over time [143]. The interaction between the lipid bilayer of the cell membrane and the cytoskeleton also strengthen over time [143]. EryP can be detected in the circulation of the late stage embryo and newborn mouse pup at very low frequency. At E15.5, the predominant b-globin gene expressed by EryP is *ε*-globin, having switched from *β*H1-globin during maturation [65]. EryP can produce up to 80–100 pg of hemoglobin per cell [108]. Three weeks after birth, levels of *ε*-globin::KGFP+ cells in the circulation reach those of the adult mouse and are most likely background [64]. It is presumed that any EryP present in the newborn pup's circulation are cleared by the spleen in a manner similar to that seen for EryD. 

## 8. Primitive versus Definitive Erythroid Cells

**Figure 3:**
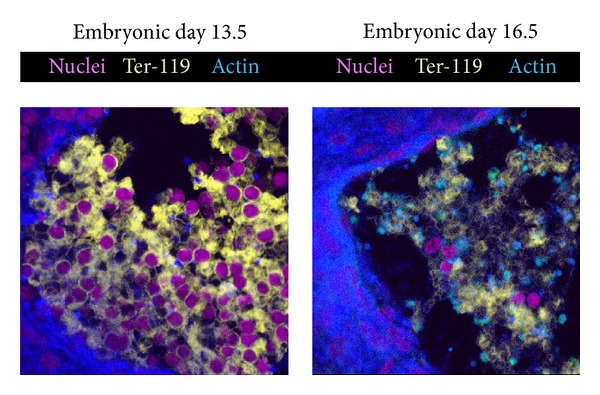
The cellular composition of the embryonic blood changes profoundly as the embryo matures. Embryonic blood cells at embryonic day E13.5 are mostly primitive nucleated (magenta) Ter-119+ (yellow) erythroblasts (left panel). Three days later, nucleated erythroid cells are very rare and have been replaced by enucleated Ter-119+ definitive erythrocytes (right panel).

A comparison of primitive and definitive erythroid cells *in situ* is shown in Figure 3. Nucleated Ter-119+ cells dominate the circulation at E13.5. However, just three days later, the circulation is primarily composed of smaller, enucleated definitive erythroid cells. How does these two arms of the erythroid system differ and what is responsible for these differences? 

### 8.1. Morphology

The classical morphological distinction between EryP and EryD is volume. Since the earliest descriptions of EryP, it was clear that they are significantly bigger than their definitive counterparts [144]. EryP are up to 80% larger than their EryD counterparts [143]. EryP isolated from the E11.5 circulation possess volumes of 400 to 750 femtolitres in contrast to 70 to 150 femtolitres, the mean cell volumes of circulating fetal liver and adult bone marrow-derived erythrocytes, respectively [26]. The cause of this difference in cell size is poorly understood. Solute carriers as well as water and ion channels can influence the cell size. 

Another classical morphological distinction between EryP and EryD is the presence of a nucleus in circulating EryP. Nucleated EryD are typically a clinical sign of poor health outcomes. Circulating nucleating erythrocytes in the adult are normally extremely low in frequency and are to be found in smokers, pregnant woman, during viral infection and following surgery. A higher level of nucleated RBCs is an indicator of poorer outcome following cardiac surgery. In contrast, EryP circulate with a nucleus from E9.0 until the fetal liver forms some 3 or 4 days later. Why EryP circulate as nucleated cells is poorly understood, however the first organ to possess large numbers of macrophages, which engulf and destroy the expelled erythroid nuclei, is the fetal liver. The placenta may be the major organ to destroy EryP nuclei in the human embryo. Therefore, there is a gap in embryonic development between the time EryP arise and the time when large numbers of macrophages are available to clear their nuclei appear. 

Marginal bands are bands of microtubules circumferentially located on the cytoplasmic side of the cell membrane. Circulating adult mammalian EryD do not possess marginal bands, however, adult erythrocytes from birds, fish, amphibians, and reptiles do contain marginal bands. Van Deurs and Behnke detected MB in EryP from the mouse however other researchers had difficulty in detecting these structures [145, 146]. Marginal bands were clearly identifiable in EryP obtained from two marsupial species [139] suggesting that EryP are likely to possess these structures. In contrast, maturing mouse definitive erythroid cells do not appear to possess marginal bands [147] (interestingly, the elliptical erythrocytes of the adult camel do possess clear marginal bands [148]). 

### 8.2. Globin Gene Expression

At the genetic level, the most prominent difference between EryP and EryD is globin gene usage [65, 149, 150]. Of the *α*-globin genes, early mouse EryP express similar amounts of the *ζ*-globin and *α*-globin [65, 151]. However, as EryP mature, theta globin expression is significantly downregulated with *α*-globin remaining the predominant *α*-chain. The mouse *β*-globin locus consists of *ε*-*β*H1-*β*1-*β*2 genes in genomic order. Early mouse EryP express *β*h1 at highest levels but proceed through a “gene switch”, and older EryP express *ε*-globin at greatest levels [65]. Adult *β*-globin is also expressed although at very low levels [65]. In contrast, EryD at these stages of development exclusively express *β*1 and *β*2 *β*-globin genes [65]. *β*-globin gene methylation is distinct in EryP from EryD, suggesting that, at least some, of the distinct expression profiles of the globin genes in EryP is due to methylation [152]. An enhancer has been linked to hyperacetylation of the *β*-globin locus in EryP. Deletion of this enhancer leads to reduced levels of expression of *ε*- and *β*H1-globins [153]. Human EryP also proceed through a globin gene switch [154]. 

What prevents expression of embryonic globin genes in definitive erythroid cells? The Sry-related homeobox transcriptional regulator Sox6 is a known repressor of embryonic globin expression. *Sox6*-deficient embryos show abnormal circulating blood morphology with nucleated cells expressing epsilon-globin [137]. The authors suggest that this is due to the expression of embryonic globins in definitive cells; however, another interpretation of the data is that the large, embryonic globin-expressing nucleated cells at E18.5 are not abnormal definitive erythroid cells but primitive erythroid cells abnormally persisting in circulation. Sox6 induces erythroid maturation in human erythroid cells [155] and is required for the differentiation of definitive erythroid cells in the bone marrow and during stress erythropoiesis [156, 157]. Loss of Sox6 may also prevent EryP from maturing and hence lead to the increase in nucleated, embryonic-globin expressing cells in the late stage embryonic bloodstream. 

### 8.3. Differences in Transcriptional Regulation

Recent transcriptome analyses of purified EryP populations allow us to dig deeper and examine the genomic differences between primitive and definitive erythropoiesis [14, 66, 158]. Kingsley and colleagues identified a number of genes expressed at profoundly different levels in these two arms of the erythroid system. Both lineages expressed common regulators such as *Klf1*, *Nfe2*, *Foxn2*, *Gata1* and *Hif1a* [66]. However, EryP were found to express several transcriptional regulators at much higher levels than EryD. These include the homeobox gene *Pbx1* (a known partner of HoxB1, HoxB7, and Meis); the forkhead transcription factor *Foxh1*; *Arid3a*, *Pdlim7*, and *Cited2* [66]. Conversely, a number of genes were expressed exclusively in the definitive erythroid lineages of the foetal liver and bone marrow. Other intriguing definitive-restricted transcription factors include *Nr3c1*, *Cebpa*, *Myb*, and *Irf9*. Nr3c1 encodes Gr1, the glucocorticoid receptor. Glucocorticoid signaling is essential to definitive erythropoiesis. The synthetic glucocorticoid dexamethasone is frequently added to cultures of definitive erythroid cells to induce proliferation of erythroid progenitors [159]. Stress erythropoiesis is defective in the absence of the glucocorticoid receptor. Polymorphisms of this gene have been linked to the rare erythroid disease Diamond-Blackfan anemia leading to a loss of responsiveness of erythroblasts to corticosteroids [160]. *Myb* is a well- characterized regulator of hematopoietic development [161]. Null mutation leads to embryonic lethality due to reduced proliferation of hematopoietic progenitors [127]. *Myb* null mutation results in defects solely within the EryD, with EryP unaffected [82]. Analysing the transcriptomes of both EryP and EryD allows the identification of distinct signaling pathways being active in these distinct erythroid cells. Definitive erythroid cells respond to Stat1 inducers and IFN*γ*, whereas inhibition of STAT3 had a profound effect on EryP-CFC but no similar effect on definitive erythroid progenitor cells [158]. To date, the microarray analyses performed have focused upon transcribed mRNA species. It would be intriguing to explore the differences between these different arms of the erythroid system at the level of noncoding RNA such as microRNA or long noncoding RNA to assess other forms of gene expression regulation. 

### 8.4. Promoter Usage Differences

EryP and EryD frequently express the same gene, though often at profoundly different levels. This suggests the presence of alternate promoter sequences driving differing levels of gene expression in each arm of the erythroid system. Two well-defined examples of this phenomenon are *Gata1* and *Scl* promoter usage. Distinct upstream promoter regions drive expression of *Gata1* within the primitive versus definitive populations. Transgenic reporter systems have been fused to distinct *Gata1* promoter sequences resulting in differential patterns of reporter gene expression [162]. Expression of LacZ specifically within the EryP was restricted to transgenic mouse lines including the “upstream activating region” of the *Gata1* locus [162]. Null mutation of this EryP-specific promoter of *Gata1* lead to the loss of expression within the EryP, and therefore an accumulation of EryP due to a lack of Gata1 to upregulate maturation gene expression [163]. A similar EryP-specific promoter was found in the *Scl* locus [164]. 

### 8.5. Cell Surface Proteins Differences

Despite the obvious differences in morphology and globin gene expression, it has been challenging to identify differences in cell surface phenotype between the two erythroid lineages. Both express Ter-119, though EryP appear to express this surface glycoprotein at lower levels than EryD [104]. The utility of the *ε*-globin::H2B-GFP transgenic reporter allowed us to specifically monitor the effects of null mutation on the primitive lineage at a time when both primitive and definitive lineages were present in the circulation. Loss of the critical erythroid transcriptional regulator *Eklf* results in embryonic lethality at E13.5 from a lack of definitive erythropoiesis and a fatal anemia. *Eklf*-deficient EryP (labeled with epsilon-GFP) showed abnormal cell morphology with ruffled cell membranes and a broad range of cell size. However, these null mutant EryP could proceed through to enucleation. Intriguingly, the loss of just one copy of *Klf1* had a profound impact on the surface membrane of EryP alone. Expression of the erythroid-specific surface antigen Ter-119 was completely abrogated in the *Klf*+/− EryP whilst remaining unaffected on the EryD present in the circulation at the same stage [104]. This gene dosage response was only observed for Ter-119 expression. *Klf1*+/− EryP did not show differences with other surface markers. Whilst some surface markers were lost from the surface of Klf1-deficient EryP, others were abnormally upregulated or maintained from earlier development stages. CD31/PECAM1 and CD41 are striking examples of this abnormal expression [104]. 

The chemokine receptor CXCR7 was reported to be expressed on primitive but not definitive erythroid cells [165]. Our research group has failed to detect CXCR7 on circulating EryP using a different monoclonal antibody (Colonne and Fraser, unpublished data). However, radio-iodinated CXCL12, a cognate ligand for CXCR7, was found to bind to EryP in a competitive binding assay [165]. Expression of CXCR7 is supported by the transcriptome data available on the erythrondb URL. Expression of this chemokine receptor was observed in mouse but not human EryP [165]. However, the upregulation of CXCR7 at the orthochromatophilic primitive erythroblast stage suggests that that CXCR7 and CXCL12 may be regulating migration of EryP to the fetal liver leading to terminal differentiation. 

Aquaporins are widely known as channels that assist in equilibrating the external concentration of solutes with the internal cytoplasmic environment by the transport of water into cells. Aquaporins may also traffic solutes such as peroxides, urea, ammonia, and glycerol as long as these solutes are not charged (charged molecules cannot be transported via aquaporins). The classical aquaporin, Aqp1, is expressed highly on adult erythrocytes and kidney proximal tubular cells. Aqp1, which primarily transports water molecules, and the glycerol transporter Aqp9 are expressed by EryD and not by EryP. In contrast, EryP preferentially express Aqp3 and Apq8, known hydrogen peroxide transporters. Indeed, this group found that EryP have higher levels of radical oxygen species (ROS) such as H2O2 in their cytoplasm [66]. The transcriptomes of the erythroid lineages compared can be explored further on a website hosted at (http://www.cbil.upenn.edu/ErythronDB/). However, why EryP require increased levels of ROS for their biological activity is unclear. 

A recent proteomics comparison of human nucleated EryP and adult red blood cells identified a number of intriguing membrane proteins uniquely expressed on EryP [166]. These include neutral amino acid transporters, unique chloride channels, binding proteins, and catalytic enzymes [166]. Differences in chloride channel and aquaporin and solute carrier expression may lead to the larger cell size observed in EryP compared to adult erythrocytes. 

## 9. Primitive Erythropoiesis in Other Vertebrates

Primitive erythroid cells have been observed in a range of vertebrate model systems including fish [167], frog [168, 169] and the chick embryo [170]. EryP have been observed in embryos from a number of different species including mouse, Syrian hamster [36, 140], Mongolian gerbil [171], rabbit, cat [172], merino sheep [173], pig, cow, and human (described in [132]). Rhesus monkey ES cells have also been reported to give rise to EryP [53]. Similar patterns of EryP development were observed in these different mammalian species including formation in the YS, circulation as large, nucleated cells, loss of the nucleoli, and condensation of the nucleus [36, 172]. 

### 9.1. Primitive Erythropoiesis in Humans

Investigating primitive erythropoiesis in the developing human embryo is extremely challenging as described by Migliaccio and colleagues [174]. Embryonic tissue is obtained from clinical abortions. These procedures are often performed after the main period of primitive erythropoiesis (from week 2 to week 8 of gestation). The tissue available from these procedures is often damaged or contaminated or not preserved within a suitable period of time, leading to poor quality material for analysis [174]. In spite of these obstacles, some research groups have bravely attempted to assess the process regulating EryP formation and expansion in the human embryos. Primitive megaloblasts have been observed in the YS of 2-week-old human embryos with circulating primitive erythroblasts clearly present from week 4 to weeks 10–12 of development. Bloom and Bartelmez performed detailed histological analyses of “young human embryos” [175]. This in-depth examination of human YS morphology and histology is accompanied by exquisite illustrations of the microscopic images by Esther Bohlman [175]. Primitive erythroblasts were clearly identifiable in the YS vasculature at week 6. Esther Bohlman's beautiful illustrations clearly show the progressive maturation of primitive erythroblasts from basophilic to polychromatophilic to orthochromatophilic, in a manner since seen in the mouse embryo [175]. Hemoglobin expression in EryP and EryD was assessed in human embryos. Circulating erythroblasts from 6 week to 10 week human embryos were found to proceed through a form of globin switching from *ζ*-*α* chain expression and from *ε*-*γ* globin gene expression as they matured [154, 174]. Electron microscopic analysis of the human embryo has explored the development of EryP in greater detail [176, 177]. 

The later stages of human EryP maturation were investigated recently with the placenta implicated as a site of terminal EryP differentiation [23]. Primitive erythroid cells were monitored by flow cytometry using DRAQ5 to detect the presence of nucleic acid, CD235 (Glycophorin A) surface expression and *ζ*-globin protein immunohistochemistry [23]. By flow cytometry, small DNA+ CD245+ free nuclei could be detected in the fetal liver and particularly in the later stage (15 week) placenta. This led the authors to assess whether the placenta is a significant site of EryP terminal maturation. The human placenta contains macrophages, which when FACS-purified, were capable of forming rosettes with EryP. Embryonic globin+ CD235+ small DNA-containing structures were observed in the villous stroma of the placenta and are presumptive free EryP nuclei. EryP were found to express the nonclassical tolerogenic surface molecule HLA-G in contrast to other hematopoietic cells present in the human embryonic circulation at the same time [178]. Whilst less research has been performed on human embryonic tissue, for clear and valid technical reasons, the patterns of EryP appearance, maturation, and terminal differentiation so profound similarities with those observed in the mouse embryo. 

### 9.2. Primitive Erythropoiesis from Human Pluripotent Stem Cell Differentiation Cultures

Differentiation of human embryonic stem (hES) cell cultures can avoid the difficulties associated with analyzing early human embryonic developmental processes. *In vitro* differentiation cultures of human ES cells can give rise to early hematopoietic progenitors including EryP-CFC [179, 180]. hES-derived EryP are derived from BL-CFC cells similar to those observed in mouse ES cell cultures. The angiotensin-converting enzyme was identified as a useful surface marker of hES-derived BL-CFC [181]. A bipotent progenitor capable of generating both megakaryocytes and EryP has also been identified [83]. Large-scale production of embryonic globin-expressing nucleated EryP from hES cells is feasible [55, 56, 182] though imbalances in hemoglobin production have been reported [183]. Erythropoietin is an essential requirement for obtaining EryP-CFC from ES cell cultures. The addition of exogenous interleukin-3 (IL-3) improved the formation of EryP cultures though neither stem cell factor (SCF) nor thrombopoietin (Tpo) had any additional impact [83]. Addition of VEGF-A165 resulted in enhanced primitive erythroid cell formation [184]. EryP can also be derived from pluripotent stem cells from patients, assisting in delineating the role of disease-related genes in human primitive erythropoiesis. ES cells derived from a blastocyst, that was later genotyped to be homozygous for sickle hemoglobin, were capable of generating apparently normal EryP even though changes in globin production were observed in EryD derived from these cells [185]. 

The advent of techniques to induce pluripotent cells from terminally differentiated cells has allowed the production of hematopoietic and more specifically EryP from iPS cells derived from patients with defined genetic disorders [186, 187]. Two recent publications showed an increase in embryonic and fetal-like erythroid cell production from iPS derived from Trisomy 21 (Down Syndrome) patient fibroblasts [188]. iPS derived from patients with polycythemia vera generated higher levels of CD235+ cells in culture than those derived from normal healthy donors [189]. 

## 10. Lessons from the Primitive Erythroid Lineage to the Study of Hematological Disease

Primitive erythropoiesis takes place solely in the YS. This would imply that this developmental process has little to do with homeostasis in the adult mammal. However, the processes regulating primitive erythropoiesis are also important in hematological disorders and disease. Many of the genes which regulate EryP formation and expansion are also involved in leukemogenesis in the child or adult. Transcriptional regulators such as *Gata1*, *Scl*, *Lmo2*, and *Ldb1* all play critical roles in EryP biology and, when expressed inappropriately, in leukemia. 

To our knowledge, only one group has offered evidence that primitive erythropoiesis can be reactivated in the adult. Infantile hemangioma is a microvascular tumor identified by excessive angiogenesis. A research group in New Zealand have postulated that the cells giving rise to these tumors are similar to the hemangioblast found in the YS. They have proffered evidence to suggest that hemangioblasts and hemogenic endothelial cells can be obtained from cultured IH samples [190]. Microvascular endothelial cells in these tumors also express EpoR and *ζ*-globin, the human embryonic *β*-globin chain [191]. While endothelial expression of EpoR does not mean that these cells are erythroid, the expression of embryonic globin is intriguing. Cultured IH cells gave rise to enucleated CD235/GpA+ cells though whether these were primitive or definitive was unclear [191]. 

Whilst it is not yet clear whether the primitive erythroid program is frequently reactivated during hematological disorders, reactivation of embryonic globin has been reported and even suggested as a possible therapy for patients with chronic hematological disorders such as sickle cell anemia. Epsilon globin expression has also been detected at birth in individuals expressing Gower 2 hemoglobin (*α*2*ε*2). Transgenic mouse models of sickle cell anemia can be induce expression of human *ε*-globin transgenes [192]. Newborn individuals with *α*-thalassemia express *ζ*-globin as this is present in Hb Portland. The K562 cell line, which is a frequently used model of definitive erythroid *in vitro* differentiation, can also express embryonic globins upon erythroid differentiation induction [193]. 

## 11. Concluding Remarks

The recent boom in research into primitive erythropoiesis has opened more areas of inquiry into intriguing first blood cells. What causes EryP to be larger than EryD? Why is primitive erythropoiesis restricted to the YS? What regulates the condensation and expulsion of the primitive erythroid nucleus? Is the primitive erythroid program reactivated in adults with hematological and vascular diseases? Though often overlooked, the first red blood cell lineage to form in the embryo is essential to embryonic survival. With the application of null mutation, transgenic reporters, and pluripotent stem cell differentiation systems, the genetic, environmental and developmental requirements of this lineage are becoming better defined. The insight gained from transcriptome analyses of these cells compared to definitive erythroid lineages, allows us to better understand what makes the dominant erythroid cell of the early embryonic circulation a unique population. 

## Figures and Tables

**Table 1:** Genes critical in primitive erythropoiesis as demonstrated by null mutation.

Gene	EryP phenotype of null mutant embryos
*Klf1/Eklf *	EryP show abnormal membranes with ruffled surfaces, abnormal expression of adhesion molecules, and loss of appropriate maturation markers.
*Klf2 *	Embryonic lethality: Irregular shape of EryP with abnormal pseudopodia present. Reduced globin gene expression.
*Klf1;Klf2 *	Klf1; Klf2 compound null mutant embryos show a more severe defect than either single null mutant.
*Runx1 *	Embryonic lethality; EryP show abnormal “dimples” in their cell membranes. EryP-CFC numbers unaffected. Ter-119 expression reduced by half.
*Gata1 *	EryP progenitors appear arrested in development and are more proliferative than wild-type progenitors.
*Gata1; Gata2 *	Compound Gata1; Gata2 null mutant mice show a more severe EryP defect than single mutants. Primitive erythropoiesis is functionally absent. No *β*H1 globin gene is detectible in compound mutant embryos.
*Sox6 *	Loss of inhibition of embryonic globin expression in EryD. Large, nucleated, embryonic globin-expressing cells present in circulation late in gestation.
*Gsn (gelsolin) *	Abnormal morphology and increase in circulating binucleated and *β*H1-expressing cells.
*miR-126 *	EBs lacking miR-126 fail to support erythropoiesis. Function is via VCAM1+ supporting mesenchymal cells.
*c-Myc *	EpoR-Cre mice used to delete c-Myc in erythroid cells die at E12.5. Primitive erythroblasts show accelerated maturation presenting as orthochromatophilic erythroblasts when wild-type animals show basophilic erythroblasts. Nuclear condensation is accelerated in c-Myc null EryP.
*Abc-me/Abcb10 *	Fail of EryP to proliferate, increased ROS and apoptosis.
*Ldb1 *	Primitive erythropoieis absent from the E9.0 YS. No erythroid cells generated from *Ldb1−/−* ES cell cultures.
*Fog1 *	EryP fail to mature and differentiate. Appear as immature megaloblastic and basophilic erythroblasts at E11.5.
*Epo Receptor *	Fewer EryP are present at E9.5.
*Brg1 *	YS devoid of EryP at E9.5 due to apoptosis.
*p400/Domino *	EryP-CFC absent.
*Mdm2 *	Mice lacking the p53-inhibitor Mdm2, but not Mdm4, develop apoptotic EryP and die at E13.5.
*Adult hemoglobin *	Deletion of both adult *α*- and *β*-globin genes leads to thalassemic EryP and embryonic lethality at E13.5.

References for each mutant mouse strain are in main body of text.

**Table 2:** Changes in surface marker expression throughout the mouse primitive erythroid lifespan.

Surface protein	Early EryP-CFC (E7.5)	EryP-CFC (E8.5)	Early circulating EryP (E9.0-11.5)	Late circulating EryP (E11.5>)	Fetal liver EryP	Primitive retics.
Flk1	++	—	—	—	ND	—
CD144/VE-cadherin	++	—	—	—	ND	—
Tie2	+++	++++	—	—	ND	ND
Endoglin	+++	++++	—	—	—	—
CD31/PECAM	++++	++++	—	—	ND	—
CD41	++++	++++	—	—	—	—
c-Kit	+++	++++	—	—	—	—
CD71	ND	ND	++++	+++	+++	+
Ter-119	—	—	++	++++	++++	+++
CD9	ND	ND	++++	++++	++++	—
CD24	ND	ND	++++	++++	ND	ND
CD55	ND	ND	++++	++++	ND	ND
CD147/Basigin	ND	ND	++++	++++	ND	++++
*a4* integrin	++++	++++	—	+	++++	—
*a5* integrin	++++	++++	—	+	++++	—
*b1* integrin	++++	++++	—	+	++++	—
CD44	++++	++++	—	+	++++	—
CXCR7	ND	ND	—	+++	ND	ND

^+^Indicate relative levels of surface expression. References included in main body of text.
